# Genetic transformation and genome editing of flax (*Linum usitatissimum* L.): current status and future perspectives

**DOI:** 10.3389/fpls.2026.1923497

**Published:** 2026-09-08

**Authors:** Timur D. Mollaev, Sergey A. Bruskin, Elena N. Pushkova, Alexey A. Dmitriev, Nataliya V. Melnikova

**Affiliations:** 1 Engelhardt Institute of Molecular Biology, Russian Academy of Sciences, Moscow, Russia; 2 Vavilov Institute of General Genetics, Russian Academy of Sciences, Moscow, Russia; 3 Moscow Center for Advanced Studies, Moscow, Russia

**Keywords:** *Agrobacterium*-mediated transformation, CRISPR/Cas, genome editing, *Linum usitatissimum* L., particle bombardment, protoplast transformation, RNPs, VIGE

## Abstract

Flax (*Linum usitatissimum* L.) is an important multipurpose crop cultivated for fiber, oil (edible and industrial), and bioactive compounds used in medicine and cosmetics, making reliable transformation methods essential for targeted product quality improvement. This review compares three classic delivery platforms (*Agrobacterium*-mediated transformation, protoplast transformation, and particle bombardment) regarding efficiency, chimerism frequency, reproducibility, and suitability for genome editing, while also discussing virus-mediated delivery as a developing alternative. Currently, *Agrobacterium*-mediated transformation of hypocotyls followed by callus induction is widely used, but untransformed escapes and chimerism complicate the production of fully transgenic plants. This issue is mitigated when anther-derived calli are used as explants instead of hypocotyls and is absent in floral dip and protoplast transformation methods. Promising genome editing approaches now target the generation of non-transgenic flax plants. Although transgene-free lines were reportedly obtained through Cas-mediated oligonucleotide-directed mutagenesis (a single-stranded oligonucleotide template combined with transient clustered regularly interspaced short palindromic repeats (CRISPR)/CRISPR-associated protein (Cas) and transcription activator-like effector nucleases (TALEN) expression) in protoplasts, the lack of data confirming the absence of transgenes undermines these findings, therefore, verification of the obtained plants is essential. In theory, ribonucleoprotein (RNP) complexes could achieve a sufficient editing outcome via particle bombardment or delivery to protoplasts. Similarly, virus-induced genome editing (VIGE) utilizing viral vectors to deliver CRISPR/Cas components is also suggested as a viable approach to generate non-transgenic genome-edited plants, which is particularly advantageous as it bypasses the highly challenging plant regeneration stage.

## 1 Introduction

Flax (*Linum usitatissimum* L., Linaceae) is an ancient annual crop exploited for its oil used in edible and industrial applications, and its valuable fiber utilized in textiles and composites (Duguid, 2010; Jhala and Hall., 2010; Muzyczek, 2020; Baley et al., 2021). Omega-3 fatty acids and lignans abundant in flax oil offer protective health benefits (Kris-Etherton et al., 2003; Kezimana et al., 2018; Joana et al., 2019; Nowak and Jeziorek, 2023). Additionally, flax is used in cosmetics and exhibits significant potential in phytoremediation (Kolodziejczyk et al., 2012; Saleem et al., 2020; Cleophas et al., 2023; Raut et al., 2025).

Targeting numerous traits for genetic modification, such as enhancing fiber properties, altering oil composition, increasing seed mass, and improving resistance to biotic and abiotic stresses, is essential to increase the commercial value of flax. Transgenic lines with some of these traits improved were obtained by researchers (Lorenc-Kukuła et al., 2005; Wróbel-Kwiatkowska et al., 2007; Lorenc-Kukuła et al., 2009; Vrbová et al., 2013). The only transgenic flax variety to ever be commercialized, CDC Triffid, was resistant to sulfonylurea herbicides (McHughen et al., 1997). Registered by Canadian scientist Alan McHughen in 1996, the variety was subsequently deregistered in 2001 due to zero-tolerance policies regarding unregulated genetically modified imports to the European Union (Ryan and Smyth, 2012). The European regulatory landscape has recently shifted, although not for transgenic flax such as CDC Triffid. Regulation (EU) 2026/1388 on plants obtained by certain new genomic techniques (NGTs) was adopted by the European Parliament and the Council in June 2026, and entered into force in July 2026, with most of its provisions applying after a 24-month transition period (European Parliament and the Council of the European Union, 2026). It creates a two-tier system whereby NGT-1 plants are treated as equivalent to conventionally bred varieties following a verification procedure. Meanwhile, more complex NGT-2 plants, as well as transgenic plants, remain under the existing genetically modified organism (GMO) regime. Importantly for flax, herbicide tolerance and the production of known insecticidal substances are explicitly excluded from the simplified NGT-1 category. This is directly relevant to the two landmark traits engineered in flax to date. Glyphosate tolerance was obtained by oligonucleotide-directed mutagenesis and therefore falls within the scope of the new Regulation, yet it would be assigned to the NGT-2 category and remain subject to the GMO regime (Sauer et al., 2016). Regarding Bt-toxin production, this trait was achieved by conventional transgenesis and is regulated as a GMO, irrespective of the new framework (Dutta et al., 2021). Together, these cases underscore that trait choice, and not only the delivery or editing method, determines the regulatory pathway.

The last methodological review on flax transformation protocols was published in 2015, whereas the most recent review on genome editing in the crop, published in 2023, lacked a detailed description of transformation methods (Ludvikova and Griga, 2015; Clemis et al., 2023). Over the past decade, however, new transformation and genome editing approaches in flax and other species have been developed, while previously established protocols have been reproduced and reevaluated. This review aims to provide an overview of three transgene delivery platforms and their application to genome editing incorporating the latest research articles.

Here, we review documented variations of *Agrobacterium*-mediated transformation, protoplast transformation, and particle bombardment applied to flax. Furthermore, flax genome editing is discussed specifically as a pathway toward generating transgene-free edited plants. Finally, we compare the transformation methodologies regarding their efficiency, reproducibility, and suitability for genome editing, concluding with an outlook for future research directions.

## 2 *Agrobacterium*-mediated transformation

### 2.1 Explant types used for *Agrobacterium*-mediated transformation of flax

*Agrobacterium*-mediated transformation of flax has been successfully performed using various explants. Hypocotyls and cotyledons have been extensively utilized for co-cultivation with *A. tumefaciens* and *A. rhizogenes*, followed by regeneration via either callus induction or direct organogenesis (Zhan et al., 1988; Lorenc-Kukuła et al., 2005; Wróbel-Kwiatkowska et al., 2007; Lorenc-Kukuła et al., 2009; Vrbová et al., 2013). Anther-derived calli were successfully transformed by *A. tumefaciens* to pyramid rust-resistance alleles in the flax genome and to alter flax seed oil composition by RNAi-mediated post-transcriptional gene silencing (Chen et al., 2008, Chen et al., 2015). Seedlings subjected to mechanical puncturing of the apical meristem yielded chimeric plants carrying β-glucuronidase reporter system after treatment with *A. tumefaciens*, bypassing regeneration from the tissue culture (Kesiraju et al., 2021). Floral dip flax transformation has emerged as a highly efficient and rapid alternative method that allows obtaining non-chimeric transformants and is also free of regeneration stage (Bastaki and Cullis, 2014, Bastaki and Cullis, 2019). It was reproduced by independent researchers resulting in a transgenic flax line expressing insecticidal crystalline protein gene – *cry1A(b)* (Dutta et al., 2021).

Both transformation and regeneration efficiencies appear to vary depending on the particular genotype (Zhan et al., 1988; Vrbová et al., 2013; Bastaki and Cullis, 2019). To date, no studies have elucidated the genetic determinants underlying this phenomenon.

### 2.2 Hypocotyls as the most used flax explants

The most widespread *Agrobacterium*-mediated flax transformation protocol was first published in 1987 and included co-cultivation of hypocotyls with *A. tumefaciens* and subsequent regeneration via a callus phase (Basiran et al., 1987). Due to its reliability, and cost-effectiveness, this protocol has become a standard in flax transformation, stimulating subsequent efforts to increase its efficiency. One of the challenges researchers have attempted to overcome is the formation of untransformed escapes and chimeric shoots from the callus under selection pressure. In critical cases, 100% of regenerated shoots were reported as escapes (Zhan et al., 1988). Similarly, another study noted that 80% of the shoots were untransformed, while 45% of the remaining shoots were verified as chimeras due to the non-Mendelian segregation in their progeny (Dong and McHughen, 1993b). This phenomenon occurs because transformed cells of a callus protect the neighboring untransformed ones from the selective agent, leading to selection inefficiency (Jordan and McHughen, 1988). To mitigate this issue, an additional round of selection by transferring transformed calli to fresh selective medium was implemented (Mlynárová et al., 1994). In addition, hypocotyl pre-culturing, preliminary epidermis removal, and prolonged co-cultivation have been utilized to optimize standard protocol efficiency (Dong and McHughen, 1993a). Sonication, as another option, also enhances transformation rates but simultaneously reduces explant viability (Beranová et al., 2008). More radical tissue culture manipulations have shown a potential pathway to overcome these limitations entirely. Research on human insulin production in flax callus demonstrated that *Agrobacterium*-inoculated hypocotyls can yield loose microcalli capable of giving rise to purely non-chimeric tissues, although whole plant regeneration was not attempted in that study (Zhao et al., 2024).

### 2.3 Alternative explants for *Agrobacterium*-mediated transformation

Alternative approaches have been developed to circumvent these limitations of hypocotyl transformation efficiency. Floral dip has been described as the simplest method for generating non-chimeric transgenic flax lines, yielding a transformation efficiency of up to 50% (Bastaki and Cullis, 2019). Additionally, the escape frequency was reduced to 27% by utilizing anther-derived calli as explants, which also facilitates the rapid generation of homozygous lines via spontaneous or artificial chromosome doubling (Chen and Dribnenki, 2007).

## 3 Protoplast transformation

Plant regeneration from protoplast cultures represents a powerful alternative capable of completely eliminating the selection challenges encountered during *Agrobacterium*-mediated transformation. Protoplasts were successfully isolated from flax cotyledons, hypocotyls, roots, and shoot tips and were subsequently transformed via polyethylene glycol (PEG)-mediated transformation (Barakat and Cocking, 1983; David et al., 1994; Roger et al., 1996; Sauer et al., 2016). Plant regeneration from protoplasts was achieved but could not be reproduced in the other study due to the recalcitrance of *L. usitatissimum* to protoplast technology, contrasting sharply with other *Linum* species (Barakat and Cocking, 1983; Ling and Binding, 1992; Ling and Binding, 1997). In the later experiment, it was shown that *L. usitatissimum* plants can actually be recovered from protoplasts when a Ca^2+^-alginate matrix is used for protoplast immobilization (Roger et al., 1996). This protocol modification allowed the regeneration of non-transgenic flax plants with precise point mutations introduced by Cas-mediated oligonucleotide-directed mutagenesis, in which a single-stranded oligonucleotide template directed the edit and transiently expressed nucleases (clustered regularly interspaced short palindromic repeats (CRISPR)/CRISPR-associated protein (Cas) and transcription activator-like effector nucleases (TALEN)) generated a double-strand break that promoted its incorporation in protoplasts (Sauer et al., 2016). The described method could be highly effective for creating non-transgenic genome-edited varieties that are in high demand globally. However, another study demonstrated that PEG-mediated protoplast transformation in *L. suffruticosum* can lead to direct gene transfer into its genome (Ling and Binding, 1997). Transgene integration into protoplast genome has also been observed in *Arabidopsis thaliana* and *Nicotiana tabacum*, with its efficiency being species-specific (Negrutiu et al., 1987; Karesch et al., 1991). Consequently, the development of edited flax via the transient expression of CRISPR/Cas or other genome editing tools in protoplasts requires rigorous verification to prove the total absence of integrated transgenes.

## 4 Particle bombardment

Among the transformation methods applied to flax, particle bombardment (biolistics) is the least frequently utilized. Nevertheless, it has proven beneficial in terms of transformation efficiency. The transformation of flax hypocotyls via biolistics demonstrated significantly lower escape and chimera frequencies compared to *Agrobacterium*-mediated hypocotyl transformation (Wijayanto and McHughen, 1999). In addition, this method is preferable for delivery of large cassettes and for multiple insertions into a target genome (Kohli et al., 2003; Farré et al., 2014; Ozyigit and Yucebilgili Kurtoglu, 2020). Biolistics is also compatible with transient expression of editing constructs and with delivery of pre-assembled ribonucleoproteins (RNPs), as demonstrated in maize and wheat, making it potentially useful for flax genome editing (Svitashev et al., 2016; Liang et al., 2017). Thus, particle bombardment can be theoretically considered a viable alternative to *Agrobacterium*-mediated transformation and protoplast genome editing. However, more experimental data are required to investigate its actual efficiency.

## 5 Flax genome editing

### 5.1 Four delivery systems for genome-editing reagents

Genome editing is a powerful approach allowing precise adjustment of crop traits to improve their economic value. An increasing number of studies now focuses on obtaining transgene-free edited crops due to stringent regulatory frameworks in many countries (Gong et al., 2021; Buchholzer and Frommer, 2023). Standard genome editing tools, including CRISPR/Cas, TALEN, and zinc finger nucleases (ZFNs), can be delivered into plant cells via four distinct systems. First, the coding sequence of an editing tool can be stably integrated into plant genome using *Agrobacterium* species, with transgene-free edited lines subsequently recovered from segregated progeny. Second, the transient expression of editing tools in protoplasts allows the direct generation of desired non-transgenic plants, although this approach requires rigorous transgene-free verification. In contrast, RNPs delivered via protoplast transformation or particle bombardment represent DNA-free editing systems that are incapable of direct gene transfer into the plant genome. Finally, virus-induced genome editing (VIGE) is a rapidly developing promising approach for transgene-free edited plant production that is discussed in detail below. These four delivery systems are orthogonal to the choice of editing reagent (e.g., CRISPR/Cas, TALEN, base or prime editor), so any reagent may in principle be combined with any delivery route.

### 5.2 History of flax genome editing

The number of studies where flax genome editing has been performed remains limited. CRISPR/Cas coding sequence was successfully delivered into the flax genome by *A. tumefaciens*, inducing visible dwarf and albino phenotypes (Wang et al., 2025).

Target design in flax is shaped by its evolutionary history: cultivated flax underwent a whole-genome duplication approximately 5–9 million years ago, so many genes are present as duplicated or homoeologous copies (Sveinsson et al., 2014). Consequently, guide RNAs must either target conserved regions shared by all copies to achieve a knockout, or exploit copy-specific polymorphisms when only one homoeolog is to be edited; this also complicates the direct transfer of protocols from diploid species. Recent chromosome-scale and telomere-to-telomere flax genome assemblies now provide the resource base for such copy-specific guide design (Lu et al., 2025; Pushkova et al., 2026).

Also, glyphosate-resistant flax lines were obtained by Cas-mediated oligonucleotide-directed mutagenesis (ODM) of two *EPSPS* paralogues, in which a single-stranded oligonucleotide served as the repair template and transiently expressed CRISPR/Cas or TALEN introduced a double-strand break that strongly enhanced its incorporation (Sauer et al., 2016). However, as it was described in the “Protoplast transformation” section, this study lacks verification to ensure the total absence of integrated transgenes. The validation may be achieved via polymerase chain reaction (PCR) and Southern Blot assay targeting specific transgene sequences. Alternatively, whole-genome sequencing (WGS) can be employed, which not only confirms the lack of foreign DNA but also allows for an analysis of potential off-target editing sites. In contrast to the other two editing systems, ZFNs have never been applied to flax and are unlikely to be adopted given the operational advantages of CRISPR/Cas.

### 5.3 Perspectives of RNPs in flax genome editing

The efficiency of genome editing has been improved using RNPs, since they act immediately after delivery and are unable to insert transgenes into a host genome (Cho et al., 2013). This approach has never been applied to flax, although its efficiency has been demonstrated in other plant species. Non-transgenic genome-edited plants were obtained via either electroporation or PEG-mediated delivery of RNPs into protoplasts of pepper, tobacco, tomato, cabbage, and raspberry (Kim et al., 2020; Lee et al., 2020; Nicolia et al., 2021; Creeth et al., 2025; Blumberg et al., 2026). Moreover, similarly to protoplasts, particle bombardment offers an opportunity to deliver pre-assembled RNPs into plant cells. The efficiency of this method has already been demonstrated in maize and wheat (Svitashev et al., 2016; Liang et al., 2017). Although protoplast cultures and particle bombardment have never been exploited for RNP-mediated flax genome editing, the growing number of studies on these two approaches, alongside the established techniques of flax regeneration, opens its potential application for the genetic improvement of flax.

### 5.4 Perspectives of VIGE in flax genome editing

*In vitro* regeneration is a critical step in the production of genome-edited plants, which demands considerable time and remains highly species-dependent. Recent studies highlight VIGE as a novel approach for producing non-transgenic edited plants that effectively bypasses the tissue culture stage, as it relies on the viral infection of grown plants. Similarly to RNP-mediated genome editing, VIGE remains unexploited in flax. Nevertheless, it has been successfully established in several other plant species (Li et al., 2021; Tamilselvan-Nattar-Amutha et al., 2023; Yoshida et al., 2024; Lee et al., 2025; Weiss et al., 2025). This approach can be executed through the co-expression of a single guide RNA (sgRNA) and the Cas encoded within a single viral vector. However, viral vectors capable of carrying such large genetic cargo are often unable to infect the meristem, thereby failing to induce heritable edits in the progeny without *in vitro* regeneration (Ma et al., 2020; Liu et al., 2023). In contrast, certain viruses naturally possess the ability to enter meristematic tissues, but their smaller size limits their cargo capacity, making them unable to accommodate the large *Cas* sequence (Steinberger and Voytas, 2025). To utilize these meristem-penetrating vectors, a two-component strategy is required, where the virus delivers only the sgRNA into Cas-transgenic plants (Li et al., 2021; Tamilselvan-Nattar-Amutha et al., 2023; Lee et al., 2025). In this case, transgene-free lines can be subsequently recovered from segregated progeny. Moreover, the fusion of plant mobility motifs, such as tRNA-like sequences and *FLOWERING LOCUS T* transcripts, to the sgRNA has also been shown to facilitate transport into the meristem and promote the generation of edited progeny, though the overall efficiency of these motifs is debatable (Ellison et al., 2020; Beernink et al., 2022).

Flax has been reported to be susceptible to Tobacco rattle virus (TRV), which is frequently utilized for VIGE due to its ability to enter the meristem and exceptionally wide host range (Zein et al., 2012; Chantreau et al., 2015). Consequently, TRV-mediated VIGE represents a potential alternative for the efficient production of non-transgenic genome-edited flax plants. By directly inoculating grown flax plants, this approach could effectively bypass the challenges associated with flax *in vitro* regeneration. However, its efficiency remains theoretical due to the absence of experimental data.

## 6 Discussion

*Agrobacterium*-mediated transformation of hypocotyls remains the most widely used method for flax, heavily optimized to increase efficiency and reduce shoot escape and chimera frequencies. Among the alternative protocols, floral dip yields complete transformants with the highest efficiency. However, its outcomes appear to be genotype-specific, and the protocol has only been reproduced by independent researchers once. Utilizing anther-derived calli as explants represents a promising alternative that lowers escape frequencies and accelerates the production of homozygous lines via chromosome doubling. Co-cultivation of seedlings with a punctured apical meristem could be another viable method since it bypasses the tissue culture stage. However, the chimera frequency was not provided in the original study, highlighting a demand for data standardization in articles on the subject. Particle bombardment is another distinct approach that also reduces escape frequencies, though its application remains constrained by the availability of specialized laboratory equipment.

For genome editing, the transient expression of CRISPR/Cas components in protoplasts followed by regeneration via callus phase is highly promising. However, the initial study utilizing this approach did not provide data confirming the absence of transgene integration, as well as the examined flax variety. With careful verification, this protoplast-based method could become a standard for transgene-free flax editing. Alternatively, methods unexploited in flax yet could be first applied to the crop. For instance, RNP-based delivery represents a completely DNA-free method where verification of non-transgenic lines is not required. Although RNPs have not yet been utilized in flax, they have demonstrated reliable efficiency in other plant species via biolistics or protoplast transfection. Similarly, VIGE has not yet been applied to flax. Nevertheless, its key advantage, the omission of tissue culture regeneration, combined with the confirmed susceptibility of flax to TRV, makes VIGE a potentially powerful tool for flax genome editing.

**Figure 1:**
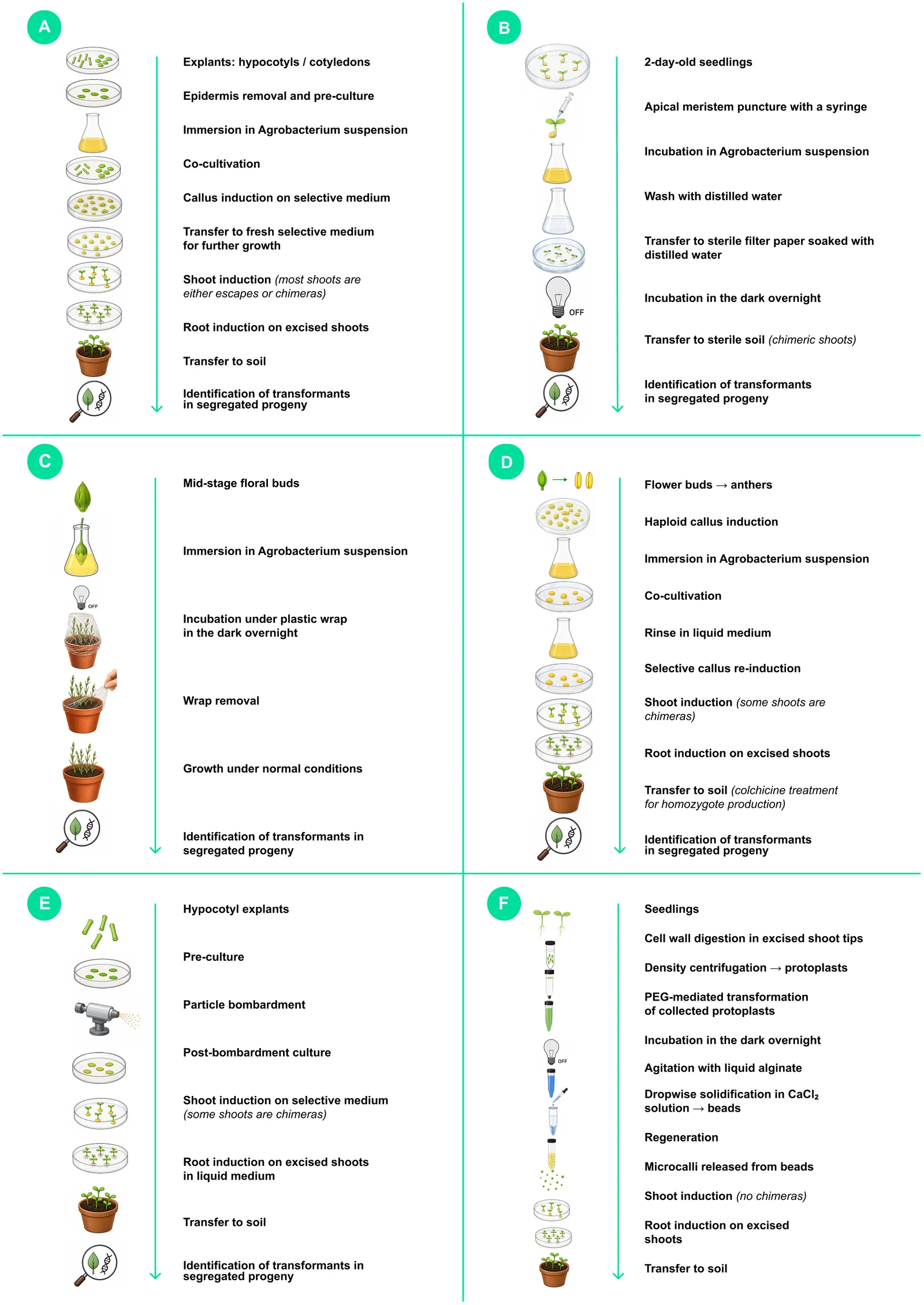
Workflows of flax transformation approaches utilized in previous articles. **(A)** *Agrobacterium*-mediated transformation of hypocotyls and cotyledons; **(B)** *Agrobacterium*-mediated transformation of punctured apical meristem; **(C)** Floral dip; **(D)** *Agrobacterium*-mediated transformation of anther-derived calli; **(E)** Particle bombardment of hypocotyls; **(F)** PEG-mediated protoplast transformation.

Potential application to genome editing and efficiency of the transformation methods that have resulted in transgenic or edited flax plants are compared in **Table 1**, and summarized workflows for these methods are illustrated in **Figure 1**. The chronological order in which flax genetic transformation and genome editing approachesappeared is illustrated in **Supplementary Figure 1**.

**Table 1:** Comparison of flax transformation approaches utilized in previous studies.

Delivery method	Target	Generation pathway	Varieties exploited	Transformation/editing efficiency*	Reproductions	Escape frequencies*	Studies	Potential application to genome editing
*Agrobacterium*-mediated transformation	Hypocotyls and cotyledons	Direct or indirect organogenesis	Numerous varieties	Up to 25%	Many	Approx. 80%	(Zhan et al., 1988; Dong and McHughen, 1993a; Mlynárová et al., 1994; Lorenc-Kukuła et al., 2005; Wróbel-Kwiatkowska et al., 2007; Lorenc-Kukuła et al., 2009; Vrbová et al., 2013)	Transgenic
	Seedlings with the punctured apical meristem	Organogenesis from apical meristem	T-397	64%	One	Not mentioned	(Kesiraju et al., 2021)	Transgenic
	Floral buds	Zygotic embryogenesis	Stormont cirrus, Bethune, Tiara	Up to 50%	A few	Zero	(Bastaki and Cullis, 2014; Bastaki and Cullis, 2019; Dutta et al., 2021)	Transgenic
	Anther-derived calli	Indirect organogenesis	Linola 1084	66%	A few	27%	(Chen et al., 2008; Chen et al., 2015)	Transgenic
Particle bombardment	Hypocotyls	Direct organogenesis	Somme	46%	One	54%	(Wijayanto and McHughen, 1999)	Transgenic, transient expression, RNPs
PEG-mediated transformation	Protoplasts	Indirect organogenesis	Not mentioned	Approx. 20%	One	Zero	(Sauer et al., 2016)	Transient expression, RNPs

The “potential application to genome editing” column indicates cross-species potential of each delivery route rather than outcomes demonstrated in flax. *Calculation methods used for transformation/editing efficiency and escape frequency vary among studies.

The bottlenecks of flax transformation mostly remain unsolved. Reliable protocols yield low transformation efficiency, while more effective ones are poorly reproduced. Regeneration remains the most restrictive step of all tissue culture-based protocols: shoot induction from callus is time-consuming and is the stage at which escapes and chimeras arise, while *L. usitatissimum* is notably recalcitrant to protoplast regeneration, which was achieved only after immobilization in a Ca^2+^-alginate matrix and has rarely been reproduced since (Roger et al., 1996; Sauer et al., 2016). Additionally, genotype dependence has been shown to influence outcomes in most transformation approaches. Future research should focus on validating high-yield protocols, specifically floral dip and transformation of anther-derived callus. To standardize reporting, articles should systematically include explant types, genotypes, time-to-production, escape/chimera frequencies, and independent replication counts. For genome-edited (as opposed to merely transformed) lines, reporting should additionally specify the editing reagent and its form (nuclease, ODM, base or prime editor, or RNP), the number of edited copies across all homoeologs, and the method and outcome of transgene-free verification (PCR, Southern blotting, or WGS) together with off-target assessment. The bottlenecks of flax genome editing itself are less evident: the editing efficiencies reported so far do not appear to be the limiting factor, and the principal constraint is instead the very small number of independent studies and the lack of transgene-free verification in them (Sauer et al., 2016; Wang et al., 2025). Thus, flax genome editing approaches, such as transient expression in protoplasts, need more reproductions, including the verification of resulting non-transgenic lines. For this purpose, WGS is an optimal method of verification since it lets researchers analyze on-target, off-targets, and transgenes at once. Alternatively, PCR and Southern blotting can be used for transgene-free verification, while on-target and predicted off-targets, can be analyzed by PCR with subsequent Sanger sequencing. Each of these approaches, however, has its own constraints. PCR is rapid and inexpensive but detects only the sequences the primers are designed against, so truncated, rearranged, or backbone-derived fragments may be overlooked, and a negative result obtained from a chimeric plant may simply reflect the sampling of untransformed tissue. Southern blotting does not require prior knowledge of the integration junctions and reports transgene copy number directly, yet it is laborious, demands microgram amounts of high-quality DNA, and has limited sensitivity for short insertions and for events present in only a fraction of cells. WGS circumvents both restrictions and resolves on-target edits, predicted and unpredicted off-target sites, and residual vector sequences in a single experiment, but it is the costliest option, requires adequate sequencing depth, and its sensitivity to low-frequency chimerism scales with coverage. For flax, the recent near-telomere-to-telomere assemblies remove the reference-genome limitation that previously restricted this approach (Lu et al., 2025; Pushkova et al., 2026). Finally, RNP delivery, as a DNA-free editing approach, and VIGE, which bypasses the tissue culture stage, are highly recommended to be first exploited in flax due to their advantages over established protocols.

## Supplementary material

Supplementary Figure 1 History of genetic transformation and genome editing of flax.
